# Effects of chloromethylisothiazolinone/methylisothiazolinone (CMIT/MIT) on Th2/Th17-related immune modulation in an atopic dermatitis mouse model

**DOI:** 10.1038/s41598-020-60966-8

**Published:** 2020-03-05

**Authors:** Han-Na Go, Seung-Hwa Lee, Hyun-Ju Cho, Jae-Rin Ahn, Mi-Jin Kang, So-Yeon Lee, Soo-Jong Hong

**Affiliations:** 1 Asan Institute for Life Sciences, University of Ulsan College of Medicine, Seoul, Korea; 2 Department of Pediatrics, International St. Mary’s hospital, Catholic Kwandong University College of Medicine, Incheon, Republic of Korea; 3 Department of Pediatrics, Environmental Health Center, Asan Medical Center, Seoul, Korea; 4 Department of Pediatrics, Childhood Asthma Atopy Center, Environmental Health Center, Asan Medical Center, University of Ulsan College of Medicine, Seoul, Korea

**Keywords:** Immunology, Inflammation

## Abstract

Exposure to chloromethylisothiazolinone/methylisothiazolinone (CMIT/MIT) has been associated with allergic contact dermatitis and occupational asthma. Despite this association however, no study has investigated the effects of CMIT/MIT exposure on the development of atopic dermatitis (AD). This study was conducted to investigate the influence of epicutaneous exposure to CMIT/MIT on AD in a mouse model and the underlying biological mechanisms. BALB/C mice were exposed to CMIT/MIT for 3 weeks and AD was developed using ovalbumin (OVA) epidermal sensitization. CMIT/MIT epicutaneous exposure in normal mice significantly enhanced AD-like phenotypes (e.g., transepidermal water loss, clinical score, total serum immunoglobulin E level and infiltration of inflammatory cells). In addition, CMIT/MIT exposure significantly augmented the mRNA expression level of T helper (Th) 2-related cytokines (thymic stromal lymphopoietin, interleukin (IL)-6 and IL-13), Th2 chemokine (chemokine (C-C motif) ligand 17) and the population of CD4^+^IL-4^+^ cells in the skin. Moreover, mice exposed to CMIT/MIT in the OVA challenge had greater AD-like phenotypes, higher IL-4 and IL-17A skin mRNA expression levels, and a larger population of CD4^+^IL-4^+^- and IL-17A^+^-producing cells in the skin-draining lymph nodes. Our current findings in a mouse model thus suggest that CMIT/MIT exposure may cause AD symptoms through the dysregulation of Th2/Th17-related immune responses.

## Introduction

Atopic dermatitis (AD) is a chronic inflammatory skin disease which manifests as eczematous skin including epidermal hyperplasia, spongiosis and immune cell infiltration of the dermis^1,2^. In recent years, and due to its high susceptibility to environmental exposure, AD has been strongly linked to a number of environmental factors, including exposure to allergens, air pollution and harmful chemical substances^3,4^. Epidemiological studies have also provided evidence for a possible relationship between environmental pollution exposure, particularly chemical substances, and the risk of AD^5–8^. Hence, the causes of the rapid increase in AD are generally thought to be environmental rather than genetic^9^.

Chloromethylisothiazolinone (CMIT) and methylisothiazolinone (MIT) are chemicals that have been widely used in biocides, paints, and cosmetics such as shampoo, body lotions and skin care products^10,11^. With the increasing uses of CMIT/MIT however, recent reports have revealed an association of these agents with allergic contact dermatitis, which acts as a sensitizer^12–14^. In addition, CMIT/MIT exposure induces a systemic allergic reaction and decrease in lung function, resulting in occupational asthma^15–17^. A recent experimental study in mice has provided evidence of a biological basis for MIT as a risk factor for allergic sensitization, as indicated by enhanced skin inflammation, immunoglobulin E (IgE) production and immune responsiveness^18^. Exposure to CMIT/MIT has the potential to enhance the sensitization to allergens, and therefore may play a crucial role in the development of allergic diseases. Despite the growing evidence for a relationship between CMIT/MIT exposure and an allergic immune response however, no previous studies have investigated an association between these chemicals and the development of AD.

Several chemical disinfectants including CMIT/MIT, polyhexamethylene guanidine phosphate and oligo (2-(2-ethoxy) ethoxyethyl guanidinium have been used in recent years in South Korea as humidifier disinfectants (HDs) because of their strong bactericidal activity and low toxicity^19^. However, HDs were later clinically confirmed in several epidemiologic studies to cause HD-associated lung injury^20–23^. In addition, people who were exposed to HDs claim to have subsequently developed allergic diseases after using HDs. Recent evidence has now shown also that HD exposure increases the risk of asthma in children^24^. However, the possible risk of AD development upon exposure to HDs is not known. We therefore aimed in our current study to investigate whether exposure to CMIT/MIT in normal mice has the ability to induce the major symptoms of AD. We also investigated whether CMIT/MIT exposure affects AD development and immune responses in an AD mouse model.

## Results

### Epicutaneous exposure of normal mice to CMIT/MIT induces AD-like skin inflammation and a systemic immune response

**Figure 1:**
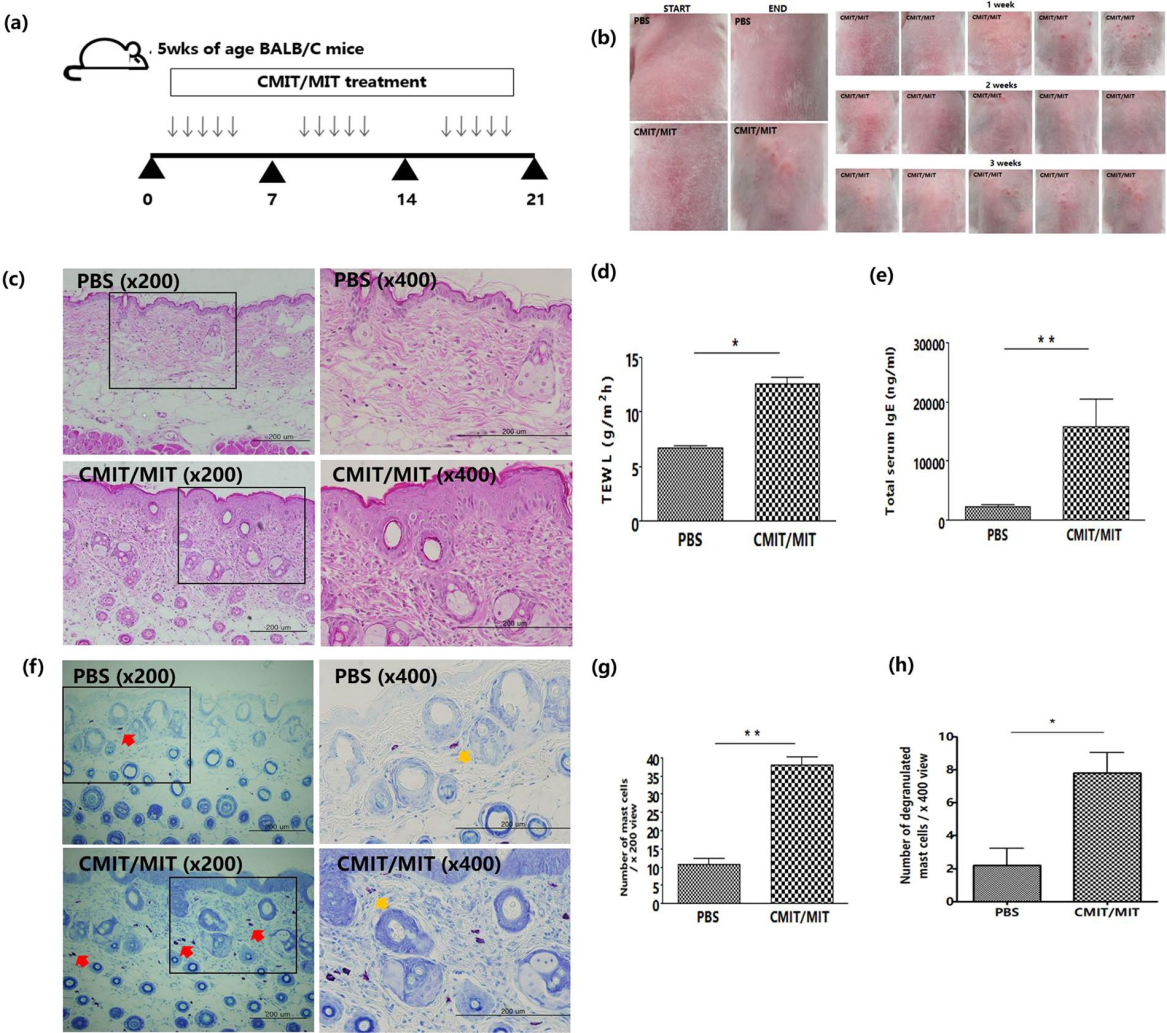
Effects of CMIT/MIT on skin lesions and histology, the epidermal permeability barrier, total IgE serum levels and the number of mast cells in the skin of normal mice. (**a**) Experimental protocol for the effects of CMIT/MIT exposure in normal mice. (**b**) Representative images of typical skin lesions. (**c**) H&E staining. (**d**) TEWL measurements. (**e**) Serum levels of total IgE determined by ELISA. (**f**) Toluidine Blue staining of skin samples. The numbers of mast cells (**g**) and degranulated mast cells (**h**) per high-power field were counted. Statistical significance was determined using a t-test. Red and yellow arrows denote the mast cells within the dermis. *P < 0.05, **P < 0.01.

**Figure 2:**
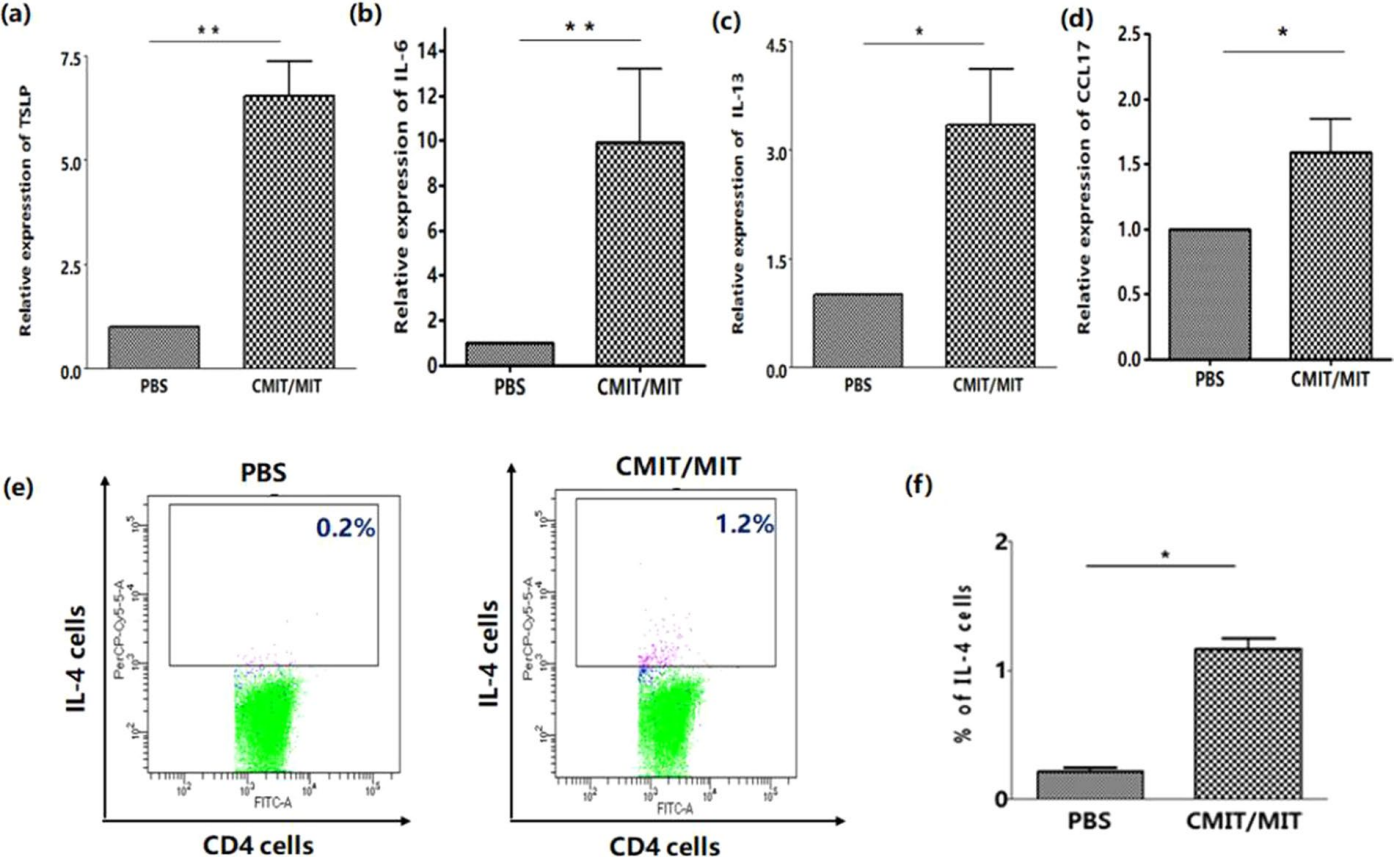
Effects of CMIT/MIT on Th2-related cytokines (TSLP, IL-6 and IL-13), a Th2-related chemokine (CCL17) and CD4^+^IL-4^+^ cell populations in normal mice. Skin mRNA levels of (**a**) TSLP, (**b**) IL-6, (**c**) IL-13 and (**d**) CCL17 were assessed by real-time PCR. (**e**,**f**) Frequency of IL-4 with CD4^+^ cells was assessed by flow cytometry in the skin-draining lymph nodes. Statistical significance was determined using a t-test. *P < 0.05, **P < 0.01.

We first investigated whether CMIT/MIT exposure induce AD-like responses in normal mice. The animals were sequentially exposed to epicutaneous CMIT/MIT over 3 weeks (Fig. 1a) which subsequently induced the formation of skin lesions (Fig. 1b). In subsequent histopathological evaluations, the mice exposed to CMIT/MIT showed a higher level of inflammatory cell infiltration and greater epidermis thickness in the skin than the control mice exposed to PBS (Fig. 1c). In addition, the mice exposed to CMIT/MIT had a higher transepidermal water loss (TEWL) (Fig. 1d) and an increase in the total serum IgE level (Fig. 1e) and mast cell number and degranulation in the skin (Fig. 1f–h) than the controls. In assessments of the immune response in these mice, the animals exposed to CMIT/MIT had a higher expression of T helper (Th) 2-related cytokines/chemokines (i.e. thymic stromal lymphopoietin (TSLP), interleukin (IL)-6, IL-13 and C-C motif chemokine (CCL)-17) in their skin (Fig. 2a–d), and greater populations of CD4+IL-4+ cells in the skin-draining lymph nodes (Fig. 2e,f) compared to the controls. However, there were no detectable IL-4 and IL-17A transcripts in the skin (data not shown). These findings demonstrated that epicutaneous exposure to CMIT/MIT in normal mice induces a phenotype that is similar to AD and involves Th2 dysfunction.

### Epicutaneous exposure to CMIT/MIT with ovalbumin (OVA) aggravates the level of skin inflammation in the AD mouse model

**Figure 3:**
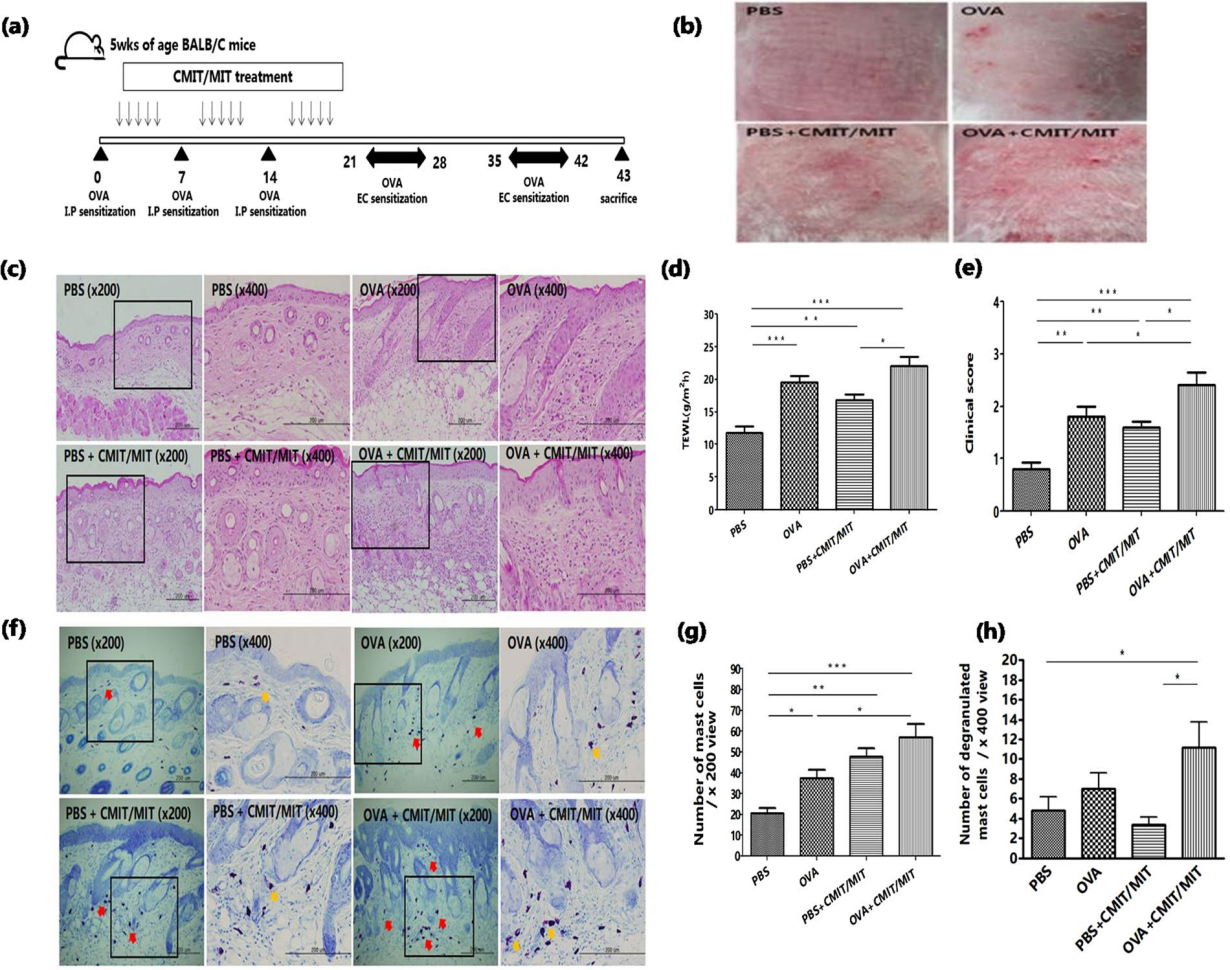
Effects of CMIT/MIT exposure in an AD mouse model. (**a**) Experimental protocol for assessing the effects of CMIT/MIT exposure in AD mice. (**b**) Representative images of typical skin lesions. (**c**) Histological analysis of H&E-stained skin tissue. (**d**) TEWL measurements. (**e**) Clinical score measurements. (**f**) Toluidine blue staining of skin samples. Numbers of mast cells (**g**) and degranulated mast cells (**h**) per high-power field were counted. Red and yellow arrows denote the mast cells within the dermis. Statistical significance was determined using ANOVA and a Newman-Keuls multiple comparison test. *P < 0.05, **P < 0.01, ***P < 0.001. I.P, intraperitoneal; EC, epicutaneous.

We investigated whether the epicutaneous exposure to CMIT/MIT during allergen sensitization would enhance the allergic AD-related consequences in the AD mouse model. The mice were exposed to CMIT/MIT during the OVA intraperitoneal sensitization phase (Fig. 3a). OVA application after CMIT/MIT exposure enhanced the formation of AD-like skin lesions (Fig. 3b). Furthermore, the mice exposed to CMIT/MIT during OVA sensitization showed a significant increase in inflammatory cell infiltration, TEWL value, clinical scores and in the number and degranulation of mast cells in skin compared to CMIT/MIT exposed and PBS control treated animals (Fig. 3c–h). In addition, mice exposed to CMIT/MIT with OVA had a greater inflammatory cell infiltration of the skin, and increased TEWL and clinical scores compared to the mice exposed to OVA alone (Fig. 3c–e), although this was not a significant difference. These findings suggested that epicutaneous exposure to CMIT/MIT during allergen sensitization enhances AD-like responses in the mouse.

### Epicutaneous exposure to CMIT/MIT with OVA increases the systemic immune response and the Th2-Th17 response in the AD mouse model

**Figure 4:**
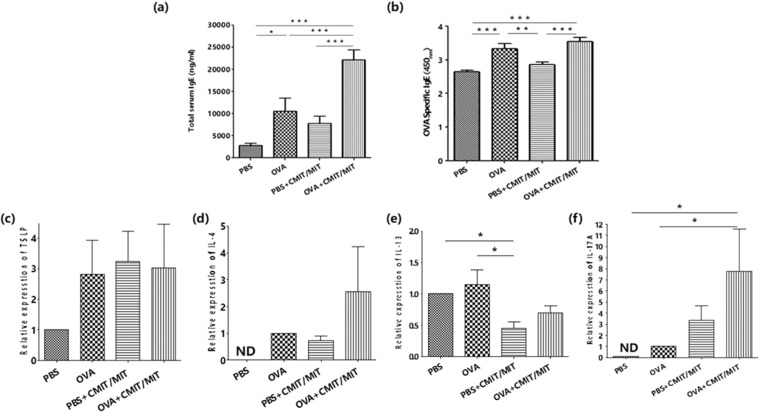
Effects of CMIT/MIT on serum IgE levels and Th2-/Th17-related responses in an AD mouse model. (**a**,**b**) ELISA determinations of (**a**) the total serum IgE levels and (**b**) the OVA-specific serum IgE levels. (**c**–**f**) Skin mRNA expression, assessed by real-time PCR, of (**c**) TSLP, (**d**,**e**) Th2-related cytokines (**d**, IL-4; **e**, IL-13) and (**f**) the Th17-related cytokine, IL-17A. Statistical significance was determined using ANOVA and the Newman-Keuls multiple comparison test. *P < 0.05, **P < 0.01, ***P < 0.001. ND, non-detection.

We next investigated whether the epicutaneous exposure to CMIT/MIT during allergen sensitization enhances the systemic immune response in AD mice. We observed that CMIT/MIT exposure during OVA sensitization significantly increased the serum total IgE levels compared with mice exposed to OVA alone (Fig. 4a). In addition, the mice exposed to CMIT/MIT with OVA showed an increased tendency toward OVA-specific IgE compared to the OVA alone animals, although this was not statistically significant (Fig. 4b).

**Figure 5:**
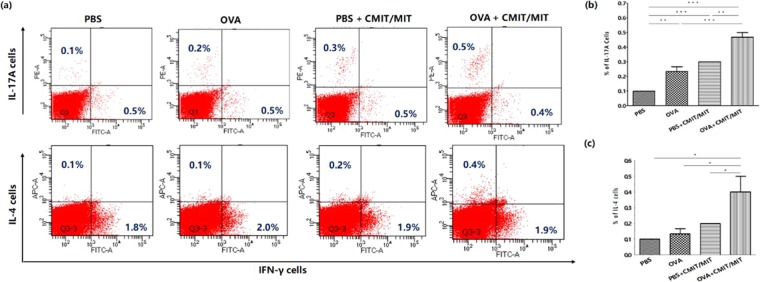
Effects of CMIT/MIT exposure on (**a**,**b**) CD4+IL-17A+ and (**a**,**c**) CD4+ IL-4+ cell populations in the skin-draining lymph nodes in the AD mouse model. Statistical significance was determined using ANOVA and the Newman-Keuls multiple comparison test. *P < 0.05, **P < 0.01, ***P < 0.001.

We next measured the AD-like immune response in both the skin and skin-draining lymph nodes in the mouse model. TSLP expression in the mouse skin was higher following exposure to CMIT/MIT with or without OVA compared to the controls (PBS alone) (Fig. 4c). Furthermore, the animals exposed to CMIT/MIT with OVA showed increased IL-4 and IL-17A expression in skin, although not IL-13, compared to the mice exposed to OVA alone (Fig. 4d–f). In addition, mice exposed to CMIT/MIT with PBS or OVA generated more CD4^+^IL-4^+^ and CD4^+^IL-17A^+^ producing cells in the skin-draining lymph nodes than the mice exposed to OVA alone (Fig. 5a–c). These findings indicated that CMIT/MIT exposure enhances the allergic systemic response and dysregulation of Th2/Th17 in the AD mouse model.

## Discussion

We have here demonstrated that repeated epicutaneous exposure to CMIT/MIT in normal mice significantly enhances AD-like phenotypes, including elevated TEWL, total serum IgE level, infiltration of inflammatory cells in the dermis, and Th2-related response in skin. Moreover, CMIT/MIT epicutaneous exposure worsened the severity of the resulting AD in subsequently OVA-induced mice, including the AD-like phenotypes and Th2/Th17-related responses in skin. Our present findings thus provide strong evidence that repeated epicutaneous exposure to CMIT/MIT may affect the development and aggravation of AD via the modulation of the immune response.

In prior clinical studies, erythema and eczema were observed during physical examinations to occur following epicutaneous airborne exposure to CMIT/MIT^10,12,25,26^. Notably also, CMIT is categorized as an extreme skin sensitizer and MIT has been proposed as a major contributor to systemic allergic reactions and skin inflammation^27–30^. Similarly, our current study using a mouse model has found that the epicutaneous exposure to CMIT/MIT induces AD-like symptoms, as reflected by an elevated TEWL, erythema, scaling, excoriation and skin inflammation. Moreover epicutaneous exposure to CMIT/MIT during allergen sensitization was further found in our analysis to enhance AD-like symptoms (TEWL, clinical score and infiltration of inflammatory cells in the dermis) compared to mice exposed to OVA alone. This further indicated that repeated epicutaneous exposure to CMIT/MIT may induce and enhance AD-like skin lesions and inflammation with/without allergen sensitization.

Additionally, our current results indicated that the total serum IgE levels and the number and degranulation extent of mast cells in the skin became significantly increased in normal mice after repeated epicutaneous exposure to CMIT/MIT. Moreover, this exposure to CMIT/MIT during allergen sensitization enhanced the total serum IgE levels and numbers of mast cells in the skin compared to the mice exposed to OVA alone. Of note in particular, the number of mast cells and the serum IgE levels in the PBS+CMIT/MIT group were still increased at 3 weeks after the last CMIT/MIT treatment compared to the PBS control group. We conducted a non-parametric t-test and found a statistical difference (P-value = 0.008) between the two groups (PBS group vs. PBS+CMIT/MIT group). The functional capacity of IgE plays an essential role in the induction and maintenance of a hypersensitivity reaction in AD. AD patients with high serum IgE levels are more likely to be involved in the development, severity, and skin barrier dysfunction of AD^31,32^. IgE molecules can trigger allergic responses by binding to their high-affinity Fc receptor (FcεRI) on mast cells^33,34^. Increased IgE concentrations enhance the IgE-dependent activity of the mast cells^35,36^. The activation of mast cells by cross-linking, through the binding of IgE to FcεRI, induces degranulation and produces Th2-type cytokines (IL-4 and IL-13) in response to IgE-dependent allergic activation. Furthermore, activated mast cells enhance their own proliferation/survival and also T-cell activation, and modulate IgE production through the secretion of survival/growth factors (IL-13 and IL-4)^37,38^. Furthermore, even in the absence of allergens, specific IgE molecules can induce some secretion of mediators through the binding to FcεRI of mast cells^35,36^. Hence, it is possible that epicutaneous exposure to CMIT/MIT contributes to an increase in IgE production and mast cell activation during allergic systemic responses and AD-like inflammation. In addition, the recruitment of mast cells and increase in serum IgE levels after CMIT/MIT exposure are likely to continue to provoke IgE-mediated systemic allergic reactions over time.

Our present study findings also revealed that Th2-related cytokines (TSLP, IL-6 and IL-13), Th2-related chemokine (CCL17) and the Th2 cell population are significantly increased immediately after exposure to CMIT/MIT, even in normal mice. In addition, CMIT/MIT epicutaneous exposure during OVA sensitization was found to increase the expression of Th2/Th17-related cytokines (IL-4 and IL-17A) and the Th2/Th17 cell population in AD mice. However, as shown in the mouse models depicted in Figs. 2 and 4, the expression of IL-13 mRNA and IL-4 and the population of IL-4 cells differ between the PBS group and CMIT/MIT treated groups. In this regard, we considered two possibilities. The first was that the sample collection times had been different. The samples for the mice in Fig. 1a were collected at 24 hours after the last CMIT/MIT treatment, and those related to Fig. 4 at 3 weeks after last CMIT/MIT treatment. The second possibility was that IL-4 and IL-13 can act on different cell types and IL-4 may drive IL-13 independent inflammation in various settings and over time^39–44^.

Interestingly, the expression of IL-4 and IL-17A in normal skin mice was not detected immediately after CMIT/MIT exposure but was increased at 3 weeks after the last CMIT/MIT treatment without OVA sensitization. Th2 cytokines (e.g., IL-4, IL-6 and IL-13) are known to play important roles in orchestrating the inflammation of AD through various routes^45–47^. Generally, allergens induce a Th2 response and subsequent Th2 cell migration by stimulating the expression of Th2 cytokines, which in turn orchestrates the allergic-related responses^48^.

Th2 cytokines are an important biomarker of prolonged eosinophilia and play a key role in orchestrating the chronic inflammation associated with AD by recruiting, activating and promoting eosinophils in the dermis. Our present study findings have demonstrated that CMIT/MIT epicutaneous exposure increase Th2-related responses. Likewise, a prior experimental study reported that the epicutaneous exposure to MIT induced an increase in T cell proliferation in mice^18^. Hence, it may be possible that exposure to CMIT/MIT in the mouse induces and enhances allergic AD-like consequences, mediated via Th2-related responses. In addition to Th2 cells, recent studies have reported that Th17 immune responses are also critical for mediating the initiation and progression of AD and differentiation of Th2 cells^45,49,50^. Several studies have further shown that Th17 responses promote tissue fibrosis, the chronicity of the inflammatory process, and AD severity and are induced by TSLP^51,52^. Our current study in the mouse has found that the expression of IL-17A and the Th17 cell population in the skin increased following CMIT/MIT epicutaneous exposure combined with OVA. These findings suggest that the exposure to CMIT/MIT during OVA sensitization can induce and enhance skin inflammation and allergic responses via Th2/Th17-mediated pathways.

Our present results have also revealed that Th2-related cytokines and Th2 cell populations are increased immediately after exposure to CMIT/MIT but that Th17 cytokines and cells are only increased at 3 weeks after the last CMIT/MIT treatment without OVA skin sensitization. This suggests that the Th2-related reactions prevail in the early stages of exposure to CMIT/MIT and the Th17-related responses prevail over time. These findings also indicate that the Th17 pathway exacerbates AD by rapidly inducing Th2 responses during OVA skin sensitization after exposure to CMIT/MIT. However, our current analysis did not investigate how increased Th17 responses after CMIT/MIT exposure directly enhances Th2 activity. Further studies are thus needed to confirm this biological relationship.

Our present study had some limitations of note. In the first instance, we could not discriminate whether allergic contact dermatitis or AD was induced by epicutaneous exposure to CMIT/MIT. Allergic contact dermatitis and AD potentially share common cellular mechanisms and it is difficult to distinguish between them in terms of a clinical diagnosis^53,54^. However, we used a previously established AD mouse model that presents with the classically characterized features of human AD i.e. a disrupted epidermal barrier function, infiltration by inflammatory cells and high serum IgE levels^55–57^. Our results indicated increases in the serum IgE levels and also the TEWL, which are considered to be features of AD after exposure to CMIT/MIT^58^. The possibility that CMIT/MIT epicutaneous exposure may affect the development of AD can thus be considered. As another limitation of our present analyses, we did not investigate direct changes to Th2-related and other biological mechanisms caused by the CMIT/MIT exposure. Further studies are needed to investigate alterations in the cellular and molecular properties resulting from CMIT/MIT treatment in the AD mouse model.

In summary, our present study has demonstrated the AD-like effects of CMIT/MIT epicutaneous exposure in normal and AD mice and suggests that the possible mechanisms involve Th2/Th17 immune responses. Further studies are needed to confirm this interaction between immune responses and biological mechanisms. Our findings suggest however that epicutaneous exposure to CMIT/MIT can affect the development of AD and can mediate Th2/Th17-related inflammation.

## Materials and Methods

### Animal experiments

Female BALB/c mice (5 weeks old, n = 5 per group) weighing 16–20 g were purchased from Orient Bio (Seongnam, Korea). All animals were housed at a temperature of 22 ± 2 °C and 40% humidity with a 12-hour light and dark cycle. All animal studies were reviewed and approved by the Animal Ethics Committee of Asan Medical Center (authorization no. 2018–14–041), and were performed in accordance with the guidelines and regulations for the Ministry of Food and Drug Safety of South Korea.

### Epicutaneous exposure to CMIT/MIT and development of AD in the mouse

In the mouse experiments, the animals were lightly anesthetized with alfaxan (Careside, Korea) and rompun (Bayer Korea, Korea) and their dorsal areas were shaved and treated with CMIT/MIT (1.13% 5-chloro-2-methyl-4-isothiazolin-3-one, 0.37% 2-methyl-4-isothiazolin-3-one, 23% inert salts; KATHON™ CG Preservative, DOW) at 0.1875 mg/kg/day, for 5 days/week over 3 weeks. Very limited information is available regarding the disease-causing possibility of CMIT/MIT, so we determined the experimental dose based on Scientific Committee on Consumer Safety (SCCS2009), report^59^, and considered the following: (1) repeated administration, (2) no observed adverse effect level (NOAEL, ≤0.104 mg/kg/day) and lowest observed adverse effect level (LOAEL, ≥0.104 mg/kg/day) (3) the concentration below threshold of skin sensitization (2.25 ug/cm^2^ in mice and 1.25 ug/cm^2^ in human) based on SCCS report contents of skin sensitization and dermal toxicity studies^59^. Generation of the AD mouse model using OVA sensitization has been previously described^55–57^. Briefly, mice were systemically sensitized via an intraperitoneal (i.p.) injection of 10 µg of chicken OVA (grade V; Sigma, St Louis, MO) and 4 mg of aluminum hydroxide (Imject Alum; Pierce, Rockford, IL) 3 times at 1week intervals. The mice were then epicutaneously (EC) sensitized by applying 100 µg OVA (grade V; Sigma, St Louis, MO) to the shaved dorsal skin of for 1week. This epicutaneous sensitization was repeated twice at 1week intervals. All mice were sacrificed at 24 hours after the final OVA sensitization.

### Assessment of TEWL and clinical scores

Clinical scores for the skin lesions in the mice were graded for erythema, scaling and excoriation as follows: 0 (no symptoms), 1 (mild), 2 (moderate), and 3 (severe)^60^. To assess epidermal permeability barrier function, the TEWL was measured using a VapoMeter (SWL-3; Delfin Technologies Ltd., Kuopio, Finland). The clinical scores and TEWL values were evaluated at baseline at the beginning of the experiment and again after each sensitization.

### Histology of the skin lesions

For the histologic evaluation of skin tissues, the area of the skin lesion was fixed in 10% formalin and then embedded in paraffin. The paraffin-embedded sections (5 μm thickness) were stained with hematoxylin and eosin (H&E) and toluidine blue (Sigma Aldrich Chemical Co. St Louis, MO) for the assessment of inflammatory cells. The total (200X magnification) and degranulated (400X magnification) mast cells per 3 high-power fields were counted and averaged per mouse (total 5 mouse) using ImageJ 1.47 software (NIH, Bethesda, MD)^61^.

### Quantitation of the serum levels of immunoglobulin

Serum samples were obtained after sacrifice and stored at −80 °C until analyzed. Total IgE levels in the serum were measured by IgE enzyme-linked immunosorbent assay (ELISA) (eBioscience, San Diego, CA). OVA-specific IgE concentrations were also measured using ELISA. Briefly, 96 well plates were coated with 100 µg/mL OVA in coating buffer (Carbonate-Bicarbonate Buffer; Sigma Chemical Co). After an overnight incubation at 4 °C, diluted serum (1:50 in carbonate-bicarbonate buffer) was added to the wells and incubated for 1 h at 37 °C. The plates were washed and 20 ng/100 *μ*ℓ anti-mouse IgE (Acris Antibodies, Herford, Germany) was added for a further 1 h at 37 °C. After additional washing, 100 µl of 3,3′,5,5′-tetramethylbenzidine (TMB) solution (Sigma Chemical Co.) was added to each well and the plate was read at 450 nm.

### Real-time PCR

Total RNA from mouse skin was extracted using an RNeasy kit (Qiagen, Valencia, CA) and cDNA was synthesized from 1 µg aliquots of these preparations using a WizScript cDNA synthesis Kit (Wizbiosolutions, Korea). qPCR was performed on an ABI 7900 system (Applied Biosystems, Piscataway, NJ). The expression level of each gene was normalized to that of glyceraldehyde 3-phosphate dehydrogenase (GAPDH).

### Flow cytometry

Mouse skin-draining lymph nodes (LNs; axillary and brachial lymph nodes) were harvested immediately after sacrifice and dissociated using a 70-um cell strainer (SPL Life Sciences, Pocheon, Korea). LN cells were cultured with 1X Protein Transport Inhibitor Cocktail (500×, eBioscience, San Diego, CA) and 1X Cell Stimulation Cocktail (500×, eBioscience) for 16 hours to stimulate and expand the T cells. Mouse interferon gamma (IFN-γ), IL-4 and IL-17A were subsequently assessed by flow cytometry^62^. The LNs were then stained using FITC-labeled anti-IFN-γ, PERCP-CY5.5-labeled anti-CD4, APC-labeled anti-IL-4, PE-labeled anti-IL-17A and the respective isotype controls (eBioscience) to analyze CD4^+^ IFN-γ, IL-4 and IL-17A expression in accordance with the mouse Th1/Th2/Th17 Phenotyping Kit general protocol (BD Biosciences, San Jose, CA). The stained cells were then analyzed by FACS Canto with BD FACSDiva 8.0.1 (BD Biosciences, Mountain View, CA).

### Statistical analysis

Comparisons between the two groups were conducted using nonparametric tests (Mann Whitney U-test), and the ANOVA and Newman-Keuls multiple comparison tests were used to assess differences in the measurements between multiple groups. Statistical analyses were performed using Graph Pad Prism 4.0 (San Diego, CA). The independent samples t-test was conducted using SPSS version 24.0 (SPSS Inc., Chicago, IL).
